# Alcohol as a Remedy for the Poison of the Rattlesnake

**Published:** 1852-11

**Authors:** Harvey Lindsly

**Affiliations:** Washington, D. C.


From the Stethoscope.

*Alcohol as a Remedy for the Poison of the Rattlesnake, by* HARVEY LINDSLY, M. D., of Washington, D. C.

I HAVE recently seen a paragraph in several newspapers, giving an account of two or three boys who were bitten by a venomous snake, and were relieved by drinking freely of brandy or whisky. This reminded me of a similar striking case, which came under my own observation, in this city, about two years since, which I have hitherto neglected to bring to the notice of the profession. It involves a question, however, of such great practical importance, that it seems to be the duty of every physician to make public all facts of which he may be cognizant, that have any bearing on the subject.

If alcohol really be a remedy, as these cases seem to prove, for the poison of the rattlesnake and other venomous snakes, (and possibly of all poisonous reptiles,) its general adoption in practice would be the means of saving annually, a great many valuable lives. It is a remedy always at hand, and one which the most ignorant can readily administer. A short time since, a physician in New York, of high standing, though attended by medical men of eminence, lost his life from the bite of a rattlesnake.

In the Autumn of 1850, a carriage drove up to my door about eight o'clock in the evening, containing four or five men, one of whom requested me to come out and see a patient they had brought for me to examine. I found him fast asleep, and, as they informed me, in a state of stupid intoxication. They said that about an hour and a half or two hours before, this man was exhibiting to his fellow soldiers, at the United States arsenal in this city, a rattlesnake which he had brought with him from Florida, when the animal suddenly thrust his head out from the box in which he was kept, and bit the man in the hand. The occurrence immediately gave rise to great confusion and alarm; and the surgeon living some two and a half miles from the post, they were at a loss to know what to do. It was soon decided, however, that they should go to the nearest grocery, and try the effects of alcohol. They accordingly gave him a large quantity of brandy, more than a pint, as they informed me. They then placed him in a carriage, and brought him to my house. Upon examination, I found him so thoroughly intoxicated, that it was impossible to rouse him; and thinking that they had already adopted the best possible remedy for the case, I directed them to take the man to his quarters again, and send for the surgeon of the post, who might resort to such local treatment as he thought best. I did not see the man again, but was informed afterward, that though very ill that night from the effects of the large quantity of brandy he had taken, he did not then or subsequently suffer in any way from the bite of the rattlesnake. Have any of your readers, Mr. Editor, met with a similar case?

September, 1852.

